# Proximal Hip Fracture: Does Canal Width Matter?

**DOI:** 10.3390/jcm14082768

**Published:** 2025-04-17

**Authors:** Maria Oulianski, Amit Sagi, Philip Rosinsky, Garrik Bilenko, Dana Avraham, Omri Lubovsky

**Affiliations:** 1 Orthopedic Department, Kaplan Medical Center, Rehovot 7639302, Israel; 2 Orthopedic Department, Barzilai Medical Center, Ashkelon 7810000, Israelomril@bmc.gov.il (O.L.)

**Keywords:** proximal femur fractures, sub-capital fractures, trochanteric fractures, radiologic evaluation of the hip, ortho-geriatric fractures

## Abstract

**Background/Objectives**: Proximal femur fractures are common in the older population and are related to bone quality. Our work evaluates bone parameters from pelvic anteroposterior (AP) radiographs in patients with trochanteric and sub-capital fractures to determine if there are predictive morphology parameters for each fracture type. **Methods**: Data from 237 medical records were extracted from patients who arrived at our hospital with trochanteric and sub-capital femoral fractures. Descriptive data and radiological evaluation of the calcar-to-canal ratio (CCR), cortical thickness index (CTI), and Dorr classification were measured by two observers and statistically evaluated. **Results**: A total of 202 patients were found to be eligible for the study. The mean patient age was 81.41 ± 7.27 years old. The mean age of the trochanteric group was significantly higher than that of the sub-capital group (*p* = 0.005). There were no statistically significant differences in gender and comorbidities. The CCR showed significance, but the CTI and Dorr classification did not show a significant difference (*p* = 0.001, *p* = 0.78, and *p* = 0.98). A high degree of reliability was shown for all measurements. The ICC for CTI and CCR was *p* = 0.791 and *p* = 0.770 (*p* < 0.001), and Cronbach’s alpha was 0.815 and 0.796, respectively. Logistic regression was found to be significant in predicting 60.4% of correct forecasts with an odds ratio of 0.011 and 95% confidence interval (*p* = 0.001). For CTI, the correct forecasting rate was 48%, with an odds ratio of 0.615 (*p* = 0.78). **Conclusions**: We found that, out of the measured parameters, the CCR stood out as important, showing that higher CCR levels are linked to an increased likelihood of trochanteric fractures.

## 1. Introduction

Proximal hip fractures are a common orthopedic trauma in elderly patients, most of whom belong to the category of ortho-geriatric patients, with ages above 65 and a mean age of 75 to 79 years [1]. In 2010, osteoporotic femoral fractures were estimated to account for 0.83% of the global burden of non-communicable diseases, leading to the loss of 5.8 million disability-adjusted life years (DALYs) [2,3]. In Europe, these fractures led to a higher number of DALYs lost compared to the DALYs lost from prevalent cancers [4]. These fractures significantly increase morbidity and mortality, mainly due to a lack of mobility [5].

In the elderly population, proximal hip fractures are considered osteoporotic fractures. Osteoporosis is a chronic, progressive skeletal disease characterized by low bone mass and deterioration of bone tissue, leading to increased fragility and a higher risk of fractures. The condition is often referred to as a “silent disease” because it typically progresses without symptoms until a fracture occurs. Osteoporosis primarily affects older adults, with postmenopausal women being at the highest risk due to the rapid loss of bone density following the decline in estrogen levels [6]. However, it can also occur in men and younger individuals, especially those with certain risk factors such as long-term corticosteroid use, smoking, excessive alcohol consumption, or a family history of the disease [7].

Common factors for increased fragility influence bone health throughout life, such as diabetes mellitus, sedentary lifestyle, malnutrition, poor physical fitness, and smoking [8].

Osteoporosis significantly worsens fracture severity and hinders proper healing, making addressing osteoporosis a critical concern. The condition can be detected through radiological images, often showing osteopenia, indicating reduced bone mass and structural decay. While increased mineralization might visually suggest denser bones, the bone density remains the same, resulting in brittle and rigid bones. Maintaining a delicate balance between bending resistance and flexibility is essential for bones to absorb energy effectively, preventing tissue damage and fractures. Excessive deformation or insufficient flexibility increases the risk of fractures by surpassing stress limits [7,9].

Occult hip fractures are those that are not immediately visible on initial plain radiographs, often requiring further imaging techniques for accurate diagnosis. These fractures are typically identified through advanced imaging such as MRI or CT scans, which are more sensitive in detecting subtle bone injuries that may not be evident on standard X-rays. Occult fractures are particularly challenging in elderly patients, as they may present with nonspecific symptoms such as pain and limited mobility, making diagnosis more difficult. Early identification and treatment are critical to prevent complications, as these fractures can lead to significant morbidity if left untreated [10].

Treatment of proximal femur fracture is almost always surgical, and the widely accepted goal is surgical treatment within 48 h to promote early mobilization and weight bearing, which helps prevent postoperative complications and ensures the best possible return to function [11,12]. The fracture pattern and location determine the surgery type and approach. The trochanteric (extracapsular) and sub-capital (intracapsular) fractures represent the most common types of proximal femoral fractures [13,14]. Trochanteric fractures can be effectively treated with internal fixation using intramedullary nails (IMNs) or sliding hip screws, while sub-capital fractures may necessitate hemiarthroplasty or total hip replacement [15,16]. Studies have shown differences in the etiologies of the two types of fractures. Most patients with bilateral proximal femoral fractures tend to have fracture locations similar to the first fracture [17]. This may indicate that proximal femoral fractures have predictive attributes of the fracture type. Possible suggestions are the mechanisms of fall, BMD, morphological features of the femur, surrounding soft tissue, and demographic fracturs [17,18,19]. Both male and female patients with trochanteric and subtrochanteric fractures are older, smaller, and have lower body weight, with lower BMD in the trochanteric area than sub-capital fractures, though BMD loss is more pronounced in women [20,21,22]. It is important to consider bone mineral density (BMD) when assessing bone loss in the trabecular part of the femur. Factors linked to sub-capital fractures include a longer femoral neck, reduced cortical thickness, an increased neck-shaft angle (NSA), and lower BMD in the cervical area of the femoral head and neck [23,24,25,26].

Plain radiographs are used as the best initial tool for assessment of the morphology of the femoral bone. In X-rays, the shafts of long bones appear tube-like, and their wall thickness (known as the cortex) can be measured against the canal diameter between the tube walls. The Dorr classification, first published in 1993 by Dorr L.D et al. [27], is a commonly used classification system for femurs today. This classification categorizes femurs as A, B, or C based on their shape and bone structure. Type A femurs have the narrowest isthmus (the narrowest part of the femoral canal), while type C femurs have the widest. The isthmus is defined as the narrowest part of the canal. The thinner the bone, the thinner the cortex, and the wider the canal’s diameter. This was primarily used to decide whether cemented or cementless implants should be used for patients undergoing total hip replacement. The initial evaluation is typically made through subjective radiologic assessment of plain radiographs by analyzing the cortices [16,27]. In a later work by Dossick et al., the width of the medullary canal and the morphological measurement of the calcar-to-canal ratio (CCR) were correlated to numeric parameters for each of the three Dorr classifications (type A ≤ 0.5, type B = 0.5−0.75, type C ≥ 0.75) [27,28,29,30,31].

This study investigates the relationship between femur wall thickness and the canal’s diameter within the cortex as demonstrated by the Dorr classification and assesses whether this measurement is consistent with bone quality. The investigators hypothesize that the measured bone parameters may be used to predict a proximal femur fracture’s type and its anatomical location.

## 2. Materials and Methods

### 2.1. Ethics

This study was approved by the institutional research committee and conducted according to the Helsinki Declaration (approval number BMC-0112-17). As this study is retrospective and did not include additional medical intervention, there was no need for patient consent. Findings were reported according to the STrengthening the Reporting of OBservational studies in Epidemiology (STROBE) checklist for retrospective cohort studies [28].

### 2.2. Study Design and Population

Data of 237 patients with either trochanteric or sub-capital femoral fractures (including both basicervical and transcervical fractures) were identified using the International Classification of Diseases, 9th edition (ICD-9) diagnosis of hip fracture (820.xx). The data were retrospectively extracted from the medical records of patients who arrived at the emergency department (ED) of a tertiary medical center. Demographic data, such as age, gender, and Charlson comorbidity score, were obtained from medical records. Only patients with plain anteroposterior (AP) radiographs of the pelvis in a correct standardized radiographic technique were eligible for the study. Radiographs were taken from the digital picture archiving and communicating system (PACS) (Kodak Carestream, Eastman Kodak Company, New York, NY, USA). All radiographs were taken upon arrival to the ED; measurements were performed on the contralateral hip (to the fractured femur). Inclusion criteria were age above 60 years old, low-energy injury after falling from a standing position, and no history of previous surgeries or evidence of pathological fractures. Reciprocally, exclusion criteria were high-energy trauma, pathological fracture, and previous surgeries of lower extremities.

### 2.3. Measurements

AP pelvic radiographs were analyzed for the following parameters: CW10, EW10 (cortical width and endosteal width 10 cm from the lesser trochanter, respectively), EWlt (endosteal width at the level of lesser trochanter), calcar-to-canal ratio (CCR), cortical thickness index (CTI), and Dorr femoral bone classification. Two independent observers (AM, PR) took measurements of the radiographs.

In a long bone X-ray, the femoral shaft looks like a tube. The wall thickness of the tube, known as the cortex, can be measured in relation to the canal diameter between the tube walls.

**Figure 1:**
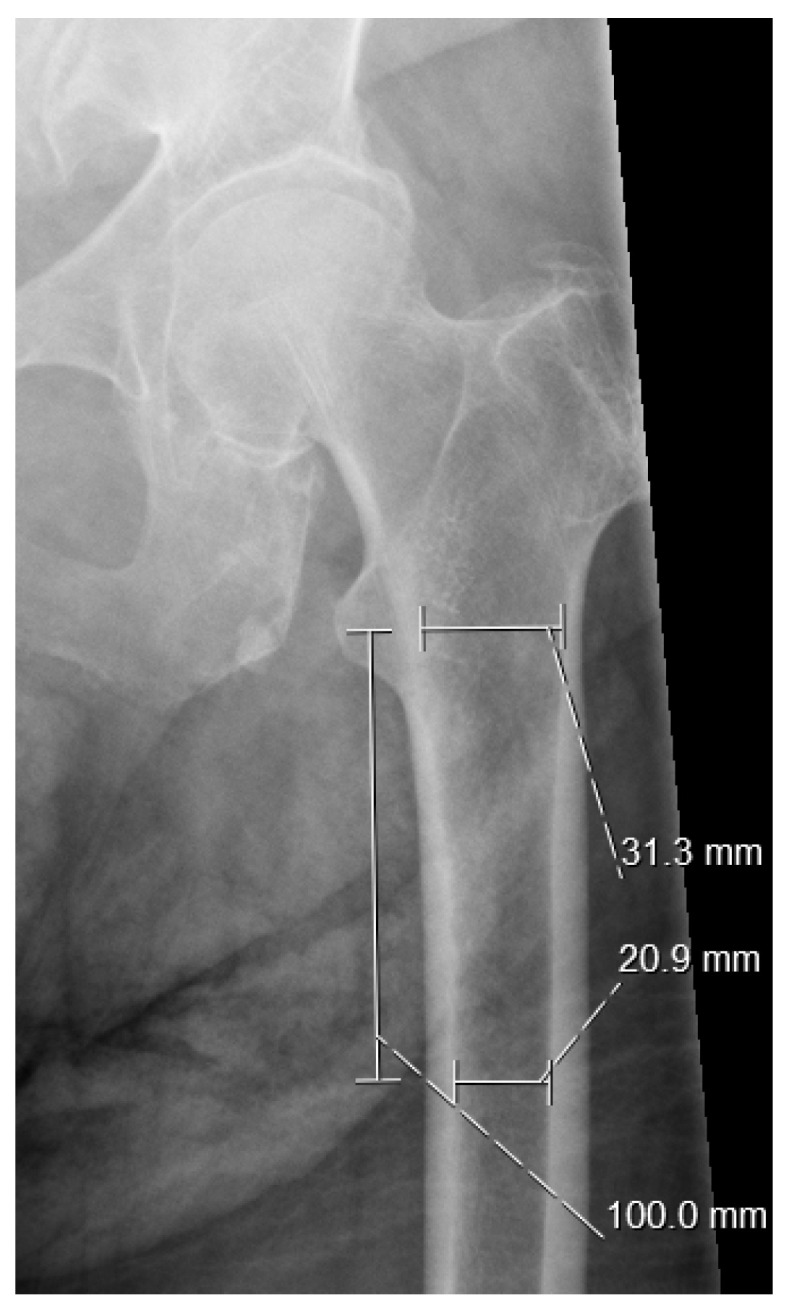
Calcar-to-canal ratio—CCR. Example: 20.9/31.3 = 0.667.

CCR is calculated as a fraction of the isthmus canal width divided by the calcar canal dimension, which represents the distance between intersection points inside the medullary canal at 3 cm and 10 cm; thus, the ratio of the calcar width to the intramedullary femoral width can be determined (Figure 1) [27].

**Figure 2:**
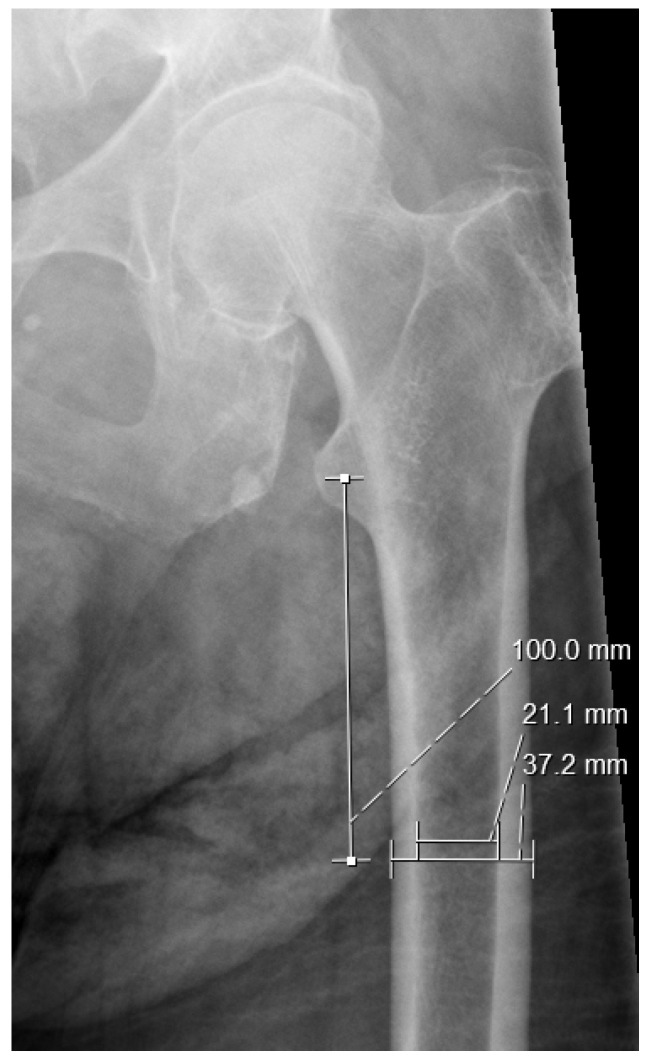
Cortical thickness index—CTI. Example: (37.2 − 21.1)/37.2 = 0.432.

CTI is the ratio of the femoral diaphyseal diameter minus the intramedullary canal diameter to the femoral diaphyseal diameter. These diameters were measured 10 cm below the midpoint of the lesser trochanter (Figure 2) [27,29].

Dorr classification is divided into three types based on the proximal femoral geometry. Type A is characterized by thick cortices that begin at the lesser trochanter distal end and thicken quickly, producing a funnel shape, thick cortical densities, and a narrow diaphyseal canal resembling a “champagne flute”. The meta-diaphyseal canal is narrow. Type B, in contrast, exhibits bone loss proximally (medial and posterior cortices) and widening of the diaphyseal canal. Type C shows a loss of the medial and posterior cortices thickness, resulting in a wide intramedullary canal, a narrow appearance of the bone cortices, and a “stovepipe” appearance. This classification concerns anatomical differences and the associated osteopenia, with Type C having lower T scores than Type A bone [27,30,31].

### 2.4. Statistical Analysis

Statistical data were analyzed by IBM SPSS Statistics for Windows (Version 25.0, Armonk, NY, USA). Continuous variables were analyzed using a two-tailed Student’s *t*-test or analysis of variance (ANOVA), as appropriate, and a logistic regression module as a prediction tool for categorical variables versus a continuous one. Statistical significance was at *p* < 0.05, with a confidence interval of 95%. All radiographic assessments were performed independently by two observers in a blind fashion. To assess interobserver reliability, the intraclass correlation coefficient (ICC) and Cronbach’s alpha were used for all categories separately.

## 3. Results

Two hundred and two patients presenting with trochanteric and sub-capital femoral fractures were eligible for the study. The average patient age was 81.41 ± 7.27 years old, and 68.81% were females. The mean age of the patients in the trochanteric group was significantly higher than that in the sub-capital group, at 82.84 ± 6.87 years compared to 80.00 ± 7.41 years, respectively (*p* = 0.005). There were no statistically significant differences in gender and Charlson comorbidity index between the two groups. As presented in Table 1, 64% of the patients with trochanteric fractures were female versus 73.5% of the patients with sub-capital fractures (*p* = 0.14). Charlson comorbidity index with a mean of 5.72 ± 1.82 (*p* = 0.16). There were no statistically significant differences in the CTI and Dorr classification between the two groups (*p* = 0.78, and *p* = 0.98, respectively). A significance was seen in the CCR (*p* = 0.001) parameters presented in Table 2.

**Figure 3:**
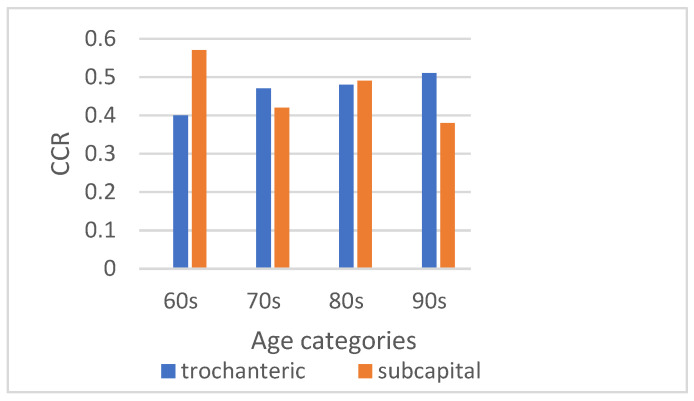
CCR by age groups.

Patients were divided according to age groups, revealing no statistical significance for the different fracture types (*p* = 0.07) and CCR (*p* = 0.121). No significance was found within the categories: the CCR for trochanteric fracture and for sub-capital fracture in different age groups was similar, with *p* = 0.12 for trochanteric and *p* = 0.8 for sub-capital (Figure 3).

A high degree of reliability was shown for all three main measurements (CTI, CCR, and Dorr classification). The average measure ICC for CTI was 0.791 with a 95% confidence interval from 0.675 to 0.859 (*p* < 0.001), while for CCR the ICC was 0.770 with a 95% confidence interval from 0.650 to 0.843 (*p* < 0.001), and Cronbach’s alpha was 0.815 and 0.796, respectively. For the Dorr classification interobserver reliability, the examination was made only on the trochanteric group with a kappa of 0.880. Subjective evaluation of the Dorr classification was tested against the calculated Dorr from the CCR according to the work of Dossicket et al. [30], revealing a kappa sum of 0.485 and 0.64 for trochanteric and 0.36 for sub-capital.

**Figure 4:**
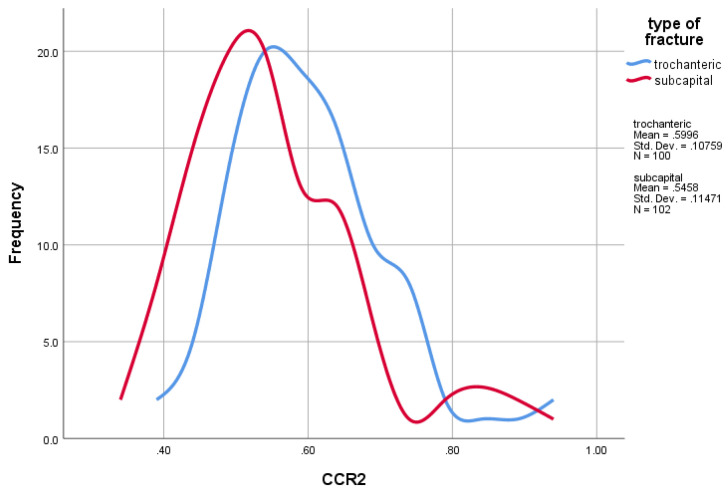
Probability of sub-capital and trochanteric fractures in the presence of CCR.

Logistic regression was completed to determine the independent prognostic value for each variable (CTI and CCR) and the possible fracture type (trochanteric and sub-capital). There was no significant association for CTI; our model predicted 48% of correct forecasting, with an odds ratio of 0.615 and 95% confidence interval from 0.02 to 17.78 (*p* = 0.78). For CCR, our model predicted 60.4%, with an odds ratio of 0.011 and 95% confidence interval from 0.001 to 0.17 (*p* = 0.001). The data suggest that, as the CCR results are lower, there is a higher probability for sub-capital fracture rather than trochanteric, as presented in Figure 4.

## 4. Discussion

This study explored the correlation between bone parameters—specifically cortical and canal width—and the types of proximal femoral fractures. These parameters were measured using pelvic AP radiographs of the uninjured hip taken upon the patient’s admission to the hospital. The primary objective was to identify potential radiographic factors that may influence the type of fracture sustained. Understanding these factors can provide valuable insight into fracture mechanisms and aid in developing prevention strategies or tailored treatment approaches.

Our findings indicate that the CTI does not significantly affect the type of fracture. However, we observed a notable correlation between the CCR and fracture type: lower CCR values were associated with sub-capital fractures, while high values tended to predict trochanteric fractures. This finding is consistent with the hypothesis that the femoral neck has different anatomical features than the trochanter area. Conversely, as the CCR increases, the structural integrity of the femoral neck is relatively preserved, making the trochanteric region more vulnerable to fracture. These findings are consistent with prior studies on rodent femoral anatomy, which demonstrate a higher cortical-to-trabecular bone ratio in the femoral neck [32]. One might claim that such a ratio contributes to the bone’s biomechanical properties, influencing load distribution and fracture susceptibility. Previous studies suggest that more than 95% of the load during weight-bearing is concentrated at the base of the neck, with approximately 50% of the load distributed across the trochanteric region and 30% impacting the sub-capital area [33]. These load-bearing characteristics highlight the biomechanical predispositions contributing to specific fracture patterns.

This study revealed that relatively younger patients were more likely to experience sub-capital fractures compared to trochanteric fractures. Among the study population, the majority of the patients (104 individuals) were in their 80s, with 56.7% presenting trochanteric fractures. However, no statistically significant difference was observed in fracture types across different age groups regarding fracture types. In 1993, a Baudoin et al. meta-analysis demonstrated that cervical fractures are more common in younger individuals, whereas trochanteric fractures predominate with advancing age; this correlates with our findings [34]. The research done by Karagas et al. in 1996 recreated similar results [35,36], reinforcing the notion that a combination of factors influences fracture patterns.

Occult proximal femoral fractures, often not visible on initial radiographs, pose a diagnostic challenge. Deleanu et al. [10] reviewed 82 cases of occult fractures, emphasizing the importance of advanced imaging techniques, such as CT or MRI, for early detection. These fractures, typically involving subtle cortical or trabecular disruptions, are often missed on conventional X-rays, especially in patients with osteoporosis. Early identification and appropriate management are crucial to prevent complications and ensure optimal outcomes.

BMD measurements at the hip have been identified as a strong predictor of hip fracture in multiple studies [37,38]; however, while it is a possible predictor of proximal femur fracture, it might not be accurate enough. Incorporating additional clinical parameters, such as medical history, smoking status, medication use, and other lifestyle factors, is essential for a comprehensive evaluation of fracture risk [34].

Our study is a non-randomized retrospective review and, as such, carries inherent limitations. We measured the unbroken femur because it is challenging to measure the broken leg in cases of preoperative displaced fractures. Although all radiographs were performed in a single center using a standardized protocol, potential biases related to imaging standards and measurement variability could theoretically impact our results. Furthermore, the unpredictable nature of falls and fractures poses a challenge for precise risk estimation. A potential limitation of this study is the variability in X-ray measurements, as results may differ depending on the person interpreting the images. Differences in experience, technique, or subjective assessment among observers could introduce inconsistencies, which may impact the reliability of the findings. Falls are inherently random events, making it nearly impossible to predict the exact timing or circumstances of a fall. This randomness introduces an additional degree of variability into the study findings, emphasizing the need for a multifaceted approach to fracture risk assessment and prevention. Future research may benefit from integrating these findings with clinical data to refine risk assessment models and optimize treatment strategies for patients at risk of proximal femoral fractures.

## 5. Conclusions

We aim to find preoperative radiographic parameters that can predict the outcome of hip fracture patients and guide us to better treatment options. Our study showed that agreement on the Dorr classification is much better when measuring canal width. The findings underscore the potential utility of the CCR in clinical decision-making and fracture risk assessment in older adults. Future studies are recommended to validate these results in larger cohorts and explore the integration of CCR measurements into routine diagnostic workflows to optimize the management of proximal femur fractures.

## Figures and Tables

**Table 1:** Sociodemographic data including age, sex, and Dorr classification types.

	Trochanteric Group (n = 100)	Sub-Capital Group (n = 102)
**Age (SD)**	82.84 (6.87)	79.99 (7.41)
Gender: **Female (n)**	64.00% (64)	73.53% (75)
**Charlson comorbidity score (SD)**	5.9 (2.00)	5.3 (1.63)
Dorr type A	19	19
Dorr type B	72	73
Dorr type C	9	10

**Table 2:** Radiological measurements evaluation on trochanteric and sub-capital fractures.

	Trochanteric, Mean (SD)	Sub-Capital, Mean (SD)	*p*-Value
CW10	32.78 (2.94)	31.30 (3.11)	0.001
EW10	16.15 (2.74)	15.51 (3.02)	0.117
EWlt	27.22 (3.61)	28.85 (4.60)	0.006
CTI	0.51 (0.07)	0.50 (0.09)	0.778
CCR	0.60 (0.11)	0.55 (0.11)	0.001

CW10 (cortical width 10 cm from the lesser trochanter), EW10 (endosteal width 10 cm from the lesser trochanter), EWlt (endosteal width at the level of the lesser trochanter), CTI (cortical thickness index), CCR (calcar-to-canal ratio).

## Data Availability

The original contributions presented in this study are included in the article. Further inquiries can be directed to the corresponding author.

## References

[1] Johnell O., Kanis J.A. (2006). An estimate of the worldwide prevalence and disability associated with osteoporotic fractures. Osteoporos. Int..

[2] Burge R., Dawson-Hughes B., Solomon D.H., Wong J.B., King A., Tosteson A. (2007). Incidence and economic burden of osteoporosis-related fractures in the United States, 2005–2025. J. Bone Miner. Res. Off. J. Am. Soc. Bone Miner. Res..

[3] Nieves J.W., Bilezikian J.P., Lane J.M., Einhorn T.A., Wang Y., Steinbuch M., Cosman F. (2010). Fragility fractures of the hip and femur: Incidence and patient characteristics. Osteoporos. Int..

[4] Borgström F., Karlsson L., Ortsäter G., Norton N., Halbout P., Cooper C., Lorentzon M., McCloskey E.V., Harvey N.C., Javaid M.K. (2020). Fragility fractures in Europe: Burden, management and opportunities. Arch. Osteoporos..

[5] Aprato A., Bechis M., Buzzone M., Bistolfi A., Daghino W., Massè A. (2020). No rest for elderly femur fracture patients: Early surgery and early ambulation decrease mortality. J. Orthop. Traumatol. Off. J. Ital. Soc. Orthop. Traumatol..

[6] Kemmler W., Bebenek M., Kohl M., von Stengel S. (2015). Exercise and fractures in postmenopausal women. Final results of the controlled Erlangen Fitness and Osteoporosis Prevention Study (EFOPS). Osteoporos. Int..

[7] Uusi-Rasi K., Karinkanta S., Tokola K., Kannus P., Sievänen H. (2019). Bone Mass and Strength and Fall-Related Fractures in Older Age. J. Osteoporos..

[8] Taylor A.J., Gary L.C., Arora T., Becker D.J., Curtis J.R., Kilgore M.L., Morrisey M.A., Saag K.G., Matthews R., Yun H. (2011). Clinical and demographic factors associated with fractures among older Americans. Osteoporos. Int..

[9] Guerado E., Cruz E., Cano J.R., Crespo P.V., Alaminos M., Sánchez-Quevedo M.d.C., Campos A. (2016). Bone mineral density aspects in the femoral neck of hip fracture patients. Injury.

[10] Deleanu B., Prejbeanu R., Tsiridis E., Vermesan D., Crisan D., Haragus H., Predescu V., Birsasteanu F. (2015). Occult fractures of the proximal femur: Imaging diagnosis and management of 82 cases in a regional trauma center. World J. Emerg. Surg..

[11] de Sire A., Invernizzi M., Baricich A., Lippi L., Ammendolia A., Grassi F.A., Leigheb M. (2021). Optimization of transdisciplinary management of elderly with femur proximal extremity fracture: A patient-tailored plan from orthopaedics to rehabilitation. World J. Orthop..

[12] Vidán M.T., Sánchez E., Gracia Y., Marañón E., Vaquero J., Serra J.A. (2011). Causes and effects of surgical delay in patients with hip fracture: A cohort study. Ann. Intern. Med..

[13] Collin P.G., D’Antoni A.V., Loukas M., Oskouian R.J., Tubbs R.S. (2017). Hip fractures in the elderly-: A Clinical Anatomy Review. Clin. Anat..

[14] Lu Y., Wang L., Hao Y., Wang Z., Wang M., Ge S. (2013). Analysis of trabecular distribution of the proximal femur in patients with fragility fractures. BMC Musculoskelet. Disord..

[15] Hopley C., Stengel D., Ekkernkamp A., Wich M. (2010). Primary total hip arthroplasty versus hemiarthroplasty for displaced intracapsular hip fractures in older patients: Systematic review. BMJ.

[16] Miller B.J., Callaghan J.J., Cram P., Karam M., Marsh J.L., Noiseux N.O. (2014). Changing trends in the treatment of femoral neck fractures: A review of the american board of orthopaedic surgery database. J. Bone Joint Surg. Am..

[17] Rotem G., Sharfman Z.T., Rath E., Gold A., Rachevsky G., Steinberg E., Drexler M., Haviv B., Amar E. (2020). Does hip morphology correlate with proximal femoral fracture type?. Hip Int. J. Clin. Exp. Res. Hip Pathol. Ther..

[18] Nasiri Sarvi M., Luo Y. (2017). Sideways fall-induced impact force and its effect on hip fracture risk: A review. Osteoporos. Int..

[19] Yamauchi K., Naofumi M., Sumida H., Fukuta S., Hori H. (2016). Comparison of morphological features in the femur between femoral neck fractures and femoral intertrochanteric fractures. Surg. Radiol. Anat..

[20] Bouxsein M.L., Szulc P., Munoz F., Thrall E., Sornay-Rendu E., Delmas P.D. (2007). Contribution of trochanteric soft tissues to fall force estimates, the factor of risk, and prediction of hip fracture risk. J. Bone Miner. Res. Off. J. Am. Soc. Bone Miner. Res..

[21] Greenspan S.L., Myers E.R., Maitland L.A., Kido T.H., Krasnow M.B., Hayes W.C. (1994). Trochanteric bone mineral density is associated with type of hip fracture in the elderly. J. Bone Miner. Res. Off. J. Am. Soc. Bone Miner. Res..

[22] Napoli N., Schwartz A.V., Palermo L., Jin J.J., Wustrack R., Cauley J.A., Ensrud K.E., Kelly M., Black D.M. (2013). Risk factors for subtrochanteric and diaphyseal fractures: The study of osteoporotic fractures. J. Clin. Endocrinol. Metab..

[23] Doblaré M., García J.M., Gómez M.J. (2004). Modelling bone tissue fracture and healing: A review. Eng. Fract. Mech..

[24] Gnudi S., Ripamonti C., Lisi L., Fini M., Giardino R., Giavaresi G. (2002). Proximal femur geometry to detect and distinguish femoral neck fractures from trochanteric fractures in postmenopausal women. Osteoporos. Int..

[25] Pande I., O’Neill T.W., Pritchard C., Scott D.L., Woolf A.D. (2000). Bone mineral density, hip axis length and risk of hip fracture in men: Results from the Cornwall Hip Fracture Study. Osteoporos. Int..

[26] Szulc P., Duboeuf F., Schott A.M., Dargent-Molina P., Meunier P.J., Delmas P.D. (2006). Structural determinants of hip fracture in elderly women: Re-analysis of the data from the EPIDOS study. Osteoporos. Int..

[27] Dorr L.D., Faugere M.C., Mackel A.M., Gruen T.A., Bognar B., Malluche H.H. (1993). Structural and cellular assessment of bone quality of proximal femur. Bone.

[28] von Elm E., Altman D.G., Egger M., Pocock S.J., Gøtzsche P.C., Vandenbroucke J.P. (2008). The Strengthening the Reporting of Observational Studies in Epidemiology (STROBE) statement: Guidelines for reporting observational studies. J. Clin. Epidemiol..

[29] Noble P.C., Alexander J.W., Lindahl L.J., Yew D.T., Granberry W.M., Tullos H.S. (1988). The anatomic basis of femoral component design. Clin. Orthop. Relat. Res..

[30] Dossick P.H., Dorr L.D., Gruen T., Saberi M.T. (1991). Techniques for preoperative planning and postoperative evaluation of noncemented hip arthroplasty. Tech. Orthop..

[31] Nakaya R., Takao M., Hamada H., Sakai T., Sugano N. (2019). Reproducibility of the Dorr classification and its quantitative indices on plain radiographs. Orthop. Traumatol. Surg. Res..

[32] Reeve J. (2017). Role of cortical bone in hip fracture. Bonekey Rep..

[33] Jordan G.R., Loveridge N., Bell K.L., Power J., Rushton N., Reeve J. (2000). Spatial clustering of remodeling osteons in the femoral neck cortex: A cause of weakness in hip fracture?. Bone.

[34] Baudoin C., Fardellone P., Sebert J.L. (1993). Effect of sex and age on the ratio of cervical to trochanteric hip fracture. A meta-analysis of 16 reports on 36,451 cases. Acta Orthop. Scand..

[35] Tanner D.A., Kloseck M., Crilly R.G., Chesworth B., Gilliland J. (2010). Hip fracture types in men and women change differently with age. BMC Geriatr..

[36] Karagas M.R., Lu-Yao G.L., Barrett J.A., Beach M.L., Baron J.A. (1996). Heterogeneity of Hip Fracture: Age, Race, Sex, and Geographic Patterns of Femoral Neck and Trochanteric Fractures among the US Elderly. Am. J. Epidemiol..

[37] Johnell O., Kanis J.A., Oden A., Johansson H., De Laet C., Delmas P., Eisman J.A., Fujiwara S., Kroger H., Mellstrom D. (2005). Predictive value of BMD for hip and other fractures. J. Bone Miner. Res. Off. J. Am. Soc. Bone Miner. Res..

[38] De Laet C., Kanis J.A., Odén A., Johanson H., Johnell O., Delmas P., Eisman J.A., Kroger H., Fujiwara S., Garnero P. (2005). Body mass index as a predictor of fracture risk: A meta-analysis. Osteoporos. Int..

